# Oral Tolerance Induced by Heat Shock Protein 65-Producing *Lactococcus lactis* Mitigates Inflammation in *Leishmania braziliensis* Infection

**DOI:** 10.3389/fimmu.2021.647987

**Published:** 2021-06-24

**Authors:** Priscila Valera Guerra, Camila Mattos Andrade, Ivanéia Valeriano Nunes, Brena Cardoso Gama, Rafael Tibúrcio, Washington Luis Conrado Santos, Vasco Ariston Azevedo, Natalia Machado Tavares, Juliana de Souza Rebouças, Tatiani Uceli Maiolii, Ana Maria Caetano Faria, Cláudia Ida Brodskyn

**Affiliations:** ^1^ Laboratório da Interação Parasita-Hospedeiro e Epidemiologia (LAIPHE) Instituto Gonçalo Moniz, Fundação Oswaldo Cruz, Salvador, Brazil; ^2^ Curso de Medicina, Centro Universitário Christus, Fortaleza, Brazil; ^3^ Laboratório de Patologia Estrutural e Molecular (LAPEM), Instituto Gonçalo Moniz, Fundação Oswaldo Cruz, Salvador, Brazil; ^4^ Departamento de Patologia e Medicina Legal Faculdade de Medicina da Universidade Federal da Bahia, Salvador, Brazil; ^5^ Departamento de Genética, Ecologia e Evolução, Instituto de Ciências Biomédicas, Universidade Federal de Minais Gerais, Belo Horizonte, Brazil; ^6^ Instituto de Investigação em Imunologia, Instituto Nacional de Ciência e Tecnologia (INCT), São Paulo, Brazil; ^7^ Instituto de Ciências Biológicas, Programa de Pós Graduação em Ciências da Saúde, Universidade de Pernambuco, Recife, Brazil; ^8^ Departamento de Nutrição, Escola de Enfermagem, Universidade Federal de Minas Gerais, Belo Horizonte, Brazil; ^9^ Departamento de Bioquímica e Imunologia, Instituto de Ciências Biológicas, Universidade Federal de Minas Gerais, Belo Horizonte, Brazil

**Keywords:** *Leishmania braziliensis*, heat shock protein 65, *Lactococcus lactis*, oral tolerance, IL-10, TLR2, TGF-β

## Abstract

Cutaneous leishmaniasis caused by *L. braziliensis* induces a pronounced Th1 inflammatory response characterized by IFN-γ production. Even in the absence of parasites, lesions result from a severe inflammatory response in which inflammatory cytokines play an important role. Different approaches have been used to evaluate the therapeutic potential of orally administrated heat shock proteins (Hsp). These proteins are evolutionarily preserved from bacteria to humans, highly expressed under inflammatory conditions and described as immunodominant antigens. Tolerance induced by the oral administration of Hsp65 is capable of suppressing inflammation and inducing differentiation in regulatory cells, and has been successfully demonstrated in several experimental models of autoimmune and inflammatory diseases. We initially administered recombinant *Lactococcus lactis* (*L. lactis*) prior to infection as a proof of concept, in order to verify its immunomodulatory potential in the inflammatory response arising from *L. braziliensis*. Using this experimental approach, we demonstrated that the oral administration of a recombinant *L. lactis* strain, which produces and secretes Hsp65 from *Mycobacterium leprae* directly into the gut, mitigated the effects of inflammation caused by *L. braziliensis* infection in association or not with PAM 3CSK4 (*N*-α-Palmitoyl-*S*-[2,3-bis(palmitoyloxy)-(2*RS*)-propyl]-L-cysteine, a TLR2 agonist). This was evidenced by the production of anti-inflammatory cytokines and the expansion of regulatory T cells in the draining lymph nodes of BALB/c mice. Our *in vitro* experimental results suggest that IL-10, TLR-2 and LAP are important immunomodulators in *L. braziliensis* infection. In addition, recombinant *L. lactis* administered 4 weeks after infection was observed to decrease lesion size, as well as the number of parasites, and produced a higher IL-10 production and decrease IFN-γ secretion. Together, these results indicate that Hsp65-producing *L. lactis* can be considered as an alternative candidate for treatment in both autoimmune diseases, as well as in chronic infections that cause inflammatory disease.

## Introduction

Leishmaniasis, the second most important protozoan infectious disease in the world, is considered endemic in 98 countries, and ranks among the 11 most prevalent parasitic diseases (1). Based on clinical manifestations, leishmaniasis is classified into tegumentary (cutaneous, mucocutaneous, disseminated and diffuse) and visceral forms [review in (2)]. Cutaneous leishmaniasis (CL), the most common form of tegumentary leishmaniasis, is characterized by a rounded ulcer with nodular or thick borders. In Brazil, the main etiological agent of CL is *Leishmania braziliensis* (3–7). Approximately 3% of patients can evolve to mucosal disease, with nasopharyngeal involvement that may cause disfiguring lesions (8). CL lesions are characterized by an exacerbated inflammatory response, with few to no parasites (9). Accordingly, the inflammatory responses mediated by proinflammatory cytokines, such as TNF, IL-1β, IL12 and IFN-γ, participate in the immunopathogenesis observed in CL (10). On the other hand, the production of IFN-γ by Th1 cells is essential to the elimination of parasites, since it activates macrophages, thereby increasing their leishmanicidal effects, which leads to the killing of *Leishmania* sp. (11). Therefore, to control the exacerbation of disease, it is necessary to modulate these inflammatory responses, while still maintaining sufficient quantities of proinflammatory mediators necessary for parasite elimination.

Our group developed a mouse model for CL caused by *L. braziliensis* that closely resembles disease of humans (12). BALB/c mice were infected in the ear dermis, provoking ulcerated dermal lesions that healed spontaneously. Histological analysis of infected ear tissue revealed the presence of mixed inflammatory infiltrate consisting of both mononuclear and polymorphonuclear cells. *In vitro* restimulation of draining lymph node cells followed by intracellular staining indicated the upregulation of IFN-γ production, as well as a higher frequency of IFN-γ-secreting CD4(+) and CD8(+) T cells (12). This model has been used in many reports in the literature aimed at furthering our understanding of CL caused by this parasite species (12).

Several studies have shown that the oral administration of antigens is one of the most effective ways of inducing regulatory T cell differentiation and peripheral tolerance. Oral tolerance (OT) is a well-known phenomenon, defined as a state of suppressed immunological reactivity against previously orally administered external antigens (13). It has been well-established that oral tolerance is more efficiently induced by the continuous low-dose feeding of antigens (14, 15). This feeding regimen has been shown to suppress inflammatory responses and induce the differentiation of regulatory T cells that produce inhibitory cytokines (16, 17).

In recent years, several types of T cells that exert regulatory functions have been identified. The principal subpopulation of regulatory T cells (Treg) responsible for self-tolerance are those that originate in the thymus, characterized by CD4+CD25+Foxp3+ (18). Other subsets of peripherally induced Tregs are also found, including CD4+CD25+Foxp3+ T cells, which originate in the intestinal mucosa (19), and CD4+ T cells, characterized by the surface expression of the latency-associated peptide (LAP), which is the N-terminal propeptide of the TGF-β precursor (20). The inhibitory functions of Tregs are mediated by different mechanisms, such as the modulation of APC function, metabolic disruption of target cells, cytotoxicity and inhibitory cytokine production (IL10, TGF-β and IL-35) (21, 22). CD4+CD25+LAP+ and CD4+CD25−LAP+ Tregs mediate suppressive cellular functions through TGF-β production (23–25).

Heat Shock Proteins (HSP), a functional class of proteins found in all living organisms, present similarities across different species. When cells are exposed to high temperatures or inflammatory stress, HSP expression becomes upregulated. Under normal conditions, lower concentrations of these proteins play a vital role in cellular housekeeping (26, 27).

Hsp60, the 60 kDa heat shock protein, seems to affect inflammation by two different mechanisms: first, as a ligand for innate immunity receptors, such as Toll-like 2 (TLR2) receptors (28), which inhibit the migration of T cells to inflammatory sites, decrease IFN-γ production and increase the release of IL-10 by T cells (29, 30); second, as an antigen recognized by receptors of adaptive immunity (31). Studies have shown that Hsp60 plays an important role in both the survival and function of regulatory CD4^+^CD25^+^Foxp3^+^ T cells (28). Oral administration of the endotoxin-free form of *Mycobacterium leprae* Hsp65, a protein similar to mammalian Hsp60, has been shown to induce oral tolerance (16, 17, 32, 33). The present study employed a novel strategy originally developed by De Azevedo et al. to deliver *Mycobacterium leprae* Hsp65 directly to the gut mucosa by continuous feeding (34). This was accomplished through the generation of a recombinant *Lactcoccus lactis (L. lactis)* strain capable of producing *M. leprae* Hsp65, which is secreted in an extracellular medium *via* a xylose-induced expression system (XIES) (34). *L. lactis* is a Gram-positive, non-invasive and non-pathogenic bacterium that does not produce endotoxins (35) and gradually releases antigens into the mucosa. This model has shown promising potential in promoting oral tolerance in humans by reproducing effects previously demonstrated in experimental models of continuous voluntary ingestion (36). The strategy has also been used in different models of autoimmune disease, such as autoimmune encephalomyelitis (16), inflammatory intestinal disease (17) and antigen-induced arthritis (32).

The oral administration of Hsp65-producing *L. lactis* prior to the infection of BALB/c mice with *L. braziliensis* resulted in a reduction in the inflammatory reaction without altering the immune response to the parasite. This first part of this study serves as a proof-of-concept that demonstrates the possibility of modulating the inflammatory response caused by *L. braziliensi*s using oral tolerance induced by recombinant *L. lactis.* Interestingly, the pre-infection administration of PAM3CSK4, a potent inflammatory agent known to promote a pronounced innate immune response (37, 38), triggered and accelerated the anti-inflammatory responses induced by Hsp65, which resulted in significantly decreased lesion severity. We chose to introduce PAM in order to test whether the induction of an inflammatory response prior to infection would amplify the immunomodulatory effects of regulatory T cells on inflammation. The effects induced by Hsp65-producing *L. lactis* were found to be associated with the production of IL-10 and the induction of CD4^+^Foxp3^+^ and CD4^+^LAP^+^ Tregs. The oral administration of recombinant *L. lactis* post-*L. braziliensis* infection also elicited a decrease in lesion size and reduced parasite load. In addition, an increase in the IL-10 and a decrease in IFN-γ production was observed, suggesting an immunomodulatory effect that led to improved healing of experimental CL lesions.

To the best of our knowledge, this is the first time that Hsp65-producing *L. lactis* has been used to attenuate infection. This experimental approach may lead to the development of novel candidates for the treatment of *L. braziliensis* infection.

## Material and Methods

### Animals

All animal procedures were approved by the Committee for Ethical Animal Use in Experimentation (CEUA N° 006/2013 IGM/Fiocruz-Bahia). Female BALB/c mice, aged 6-8 weeks, were supplied by the Animal Care Facility of the Gonçalo Moniz Institute/Fiocruz-Bahia (IGM-FIOCRUZ) in Salvador, Brazil. Mice were kept under specific pathogen-free conditions.

### Generation of Hsp65-*L. lactis*

As previously described by De Azevedo et al. (34), a recombinant *L. lactis* strain (NCDO2118) capable of secreting *M. leprae* Hsp65, was generated using a xylose-inducible expression system (XIES). The constructed vector (pSEC:hsp65) directed the expression of Hsp65 into extracellular medium. *L. lactis* NCDO2118 harboring an empty vector (pXylT : SEC without hsp65) was used as a negative control in all experiments. Dr. Faria provided these bacteria strains for our study.

### Bacterial Strain Growth and Xylose Induction

*L. lactis* NCDO2118 strains were grown in Difco M17 broth, supplemented with 0.5% glucose (GM17) or 1% xylose (XM17), at 30°C in the absence of agitation. When necessary, chloramphenicol (Cm) (10 µg/ml) was added to the medium. On the first day, a single colony of recombinant *L. lactis* harboring an empty vector (pXylT : SEC) or recombinant *L. lactis* NCDO2118 (pXylT : SEC:hsp65) was grown in 5 ml of GM17 containing chloramphenicol (Cm) (10 µg/ml). On the second day, after the culture was allowed to grow overnight, it was diluted 1:10,000 in fresh 1% xylose M17 (XM17) supplemented with Cm (10 µg/ml) to induce the expression of the *M. leprae hsp65* gene. Cultures were ready for administration on the third day, after achieving an optical density of 2.0 at a wavelength of 600 nm, corresponding to 2.5 × 10^8^ CFU/ml.

### *L. lactis* Administration

For four days, either prior to or 4 weeks after infection with *L. braziliensis*, BALB/c mice were continuously fed either water (Lb), medium (GM17) with empty-vector-bearing *L. lactis* (Empty) or medium XM17 containing *M. leprae*-Hsp65-producing *L. lactis* (HSP). Fresh *L. lactis* culture was offered to mice daily. Assuming that each mouse consumed approximately 5 ml of culture per day (data not shown), corresponding to 7 µg/ml (34) of *M. leprae* Hsp65, we estimated the total daily dose of *M. leprae* Hsp65 to be around 35 µg per mouse.

### Parasites

The *L. braziliensis* promastigotes (strain MHOM/BR/01/BA788) used in experimentation were cultured in Schneider’s medium (Sigma Chemical Co., St Louis, MO, USA) supplemented with 10% inactivated fetal bovine serum (Gibco, USA), L-glutamine (2 mM), penicillin (100 U/ml) and streptomycin (100 µg/ml), at 23°C for approximately 7 days to enable parasites to reach stationary phase.

### Experimental Infection, Lesion Measurement, and Parasite Load Estimation

Five experimental groups of mice were formed, firstly considering those that received water (Lb), medium GM17 with empty-vector-bearing *L. lactis* (Empty) and medium XM17 with Hsp65-producing *L. lactis* (HSP). At 48 hours prior to infection, an intraperitoneal dose of 20µg PAM3CSK4 (InvivoGen) was administered in two additional groups: one that received medium with Hsp65-producing *L. lactis* (HSP+PAM) and another that received medium alone (PAM). Ten days after oral administration with *L. lactis* strains, all mice were challenged in the ear dermis with stationary-phase metacyclic *L. braziliensis* promastigotes (2 x 10^5^ parasites in 10µl of sterile saline). Metacyclic forms were purified using *Bauhinia purpurea* lectin [as previously described by Pinto-da-Silva et al. (39)]. In the case of treatment post-infection, at 4 weeks after infection mice received empty or recombinant *L. lactis* for four days. Ear lesion thickness was monitored weekly using a digital caliper (Thomas Scientific, USA). Parasite load was determined individually using a quantitative limiting dilution assay as previously described (40).

### Cell Cultures and Cytokine Assay

Ear draining lymph nodes from each mouse were carefully and aseptically collected, macerated individually using a cell strainer and the cell suspensions were centrifuged at 400 x g for 10 minutes at 4°C. Cells were counted, adjusted to a concentration of 1x10^6^ cells/well and cultured in complete RPMI [RPMI 1640 supplemented with 10% inactivated fetal bovine serum (Sigma-aldrich), 2 mM/ml-glutamine, 100 µl/ml penicillin, 100 µg/ml streptomycin (Sigma-aldrich)] stimulated or not with live stationary-phase *L. braziliensis* promastigotes at a ratio 1:5 parasites for 72h at 37°C under 5% CO_2._ Supernatants were collected and levels of IL-10, IFN-γ, IL-4 and TGF-β were determined by ELISA using a commercial kit (Ebioscience).

### Flow Cytometry for Cell Characterization

Cell suspensions from ear draining lymph nodes were obtained as described above, then stimulated with *L. braziliensis*, or with soluble anti-CD3 and anti-CD28, which was used as an internal control for our flow cytometry analysis or left unstimulated for 18h at 37°C under 5% CO_2_. Cells were washed and stained using anti-CD4 (PerCyP 5.5), anti-LAP (TGF-β1) (PeCy7 and Alexa Fluor 405- eBioscience) and fixable viability dye (APC-Cy7). Cells were then washed and pre-incubated with a fixation/permeabilization solution (eBioscience). For intracellular staining, anti-FoxP3 (PE) was used. Flow cytometric analysis was performed on a FACS Fortessa (FACS, BD^®^ Biosciences) using FlowJo software (Tree Star Inc.). Each mouse was analyzed individually in all four experiments.

### Histopathology

Mouse ears from all experimental groups were fixed in 10% formaldehyde. Samples were processed and embedded in paraffin to obtain 5-µm thick sections, followed by staining with hematoxylin and eosin (H&E). Using an optical microscope (Olympus), all sections were evaluated for histopathological changes, including inflammation intensity and the presence of inflammatory cell infiltrate. Scores for each parameter were determined according to intensity: 0 (absence), 1 (presence of 1–25%), 2 (presence of 25-50%) and 3 (> 50%). Healthy animal ears were used as a negative control.

### *In Vitro* Experimentation

To obtain bone marrow-derived murine macrophages, mice were euthanized and their tibias and femurs were collected. After removing the epiphyses from bones, a syringe filled with sterile RPMI 1640 (Sigma-Aldrich) culture medium was used to extract bone marrow cells. After centrifugation, (400 x g for 10 minutes) the supernatant was discarded and the pellet was resuspended in 6 ml of culture medium [RPMI 1640 supplemented with 20% inactivated fetal bovine serum (Sigma-aldrich), 2 mM/ml-glutamine, 100 µl/ml penicillin, 100 µg/ml streptomycin (Sigma-aldrich) and 30% of L929 culture supernatant] and then plated in Petri dishes. Cells were incubated for seven days at 37°C under 5% CO_2_ until differentiating into macrophages. Next, macrophages were removed using ice and a cellstripper. Finally, macrophages were counted and adjusted to 1x10^6^/ml and incubated on 24 well-plates overnight.

Macrophages derived from the bone marrow of BALB/c mice were infected for 24 hours with *L. braziliensis* promastigotes at a ratio of 1:10 parasites. Co-culturing was performed, involving the infected macrophages, cells obtained from the ear draining lymph nodes of animals 10 weeks after infection and cells from the mesenteric lymph nodes of animals that received Hsp65-producing *L. lactis* (collected after 10 days of treatment). All cells were incubated in the presence or absence of anti-IL-10 at concentration of 4µg/ml, anti-LAP 4µg/ml (TGF- β1) and anti-TLR2 3µg/ml (CD282) (Biolegend) at 37°C under 5% CO_2_. All concentrations were adjusted according to the manufacturer’s data sheet. An IgG1 isotype (4µg/ml) was also employed as a negative control. Supernatants were collected after 72h of culture and levels of IFN-γ and IL-10 were determined by ELISA using commercial kits (Ebioscience).

### Statistical Analysis

Our results were submitted to D’Agostino-Pearson normality testing to verify data distribution. All statistical analyses were performed using GraphPad Prism v.5 Software (San Diego, CA, USA). To compare parametric data between groups, One-Way Analysis of Variance (ANOVA) with Tukey’s post-test was used, while Krukal-Wallis with Dunn’s post-test was employed for nonparametric data. To evaluate disease burden in each mouse, ear thickness and parasite loads were recorded weekly in all treated and control mice following challenge. These parameters were plotted individually for experimental and control mice throughout the course of infection. Disease burden was calculated as the Area Under the Curves (AUC) in plots of ear thickness and parasite load. AUC was calculated using measurement data from ear thickness and parasite load in each group. AUC values were then compared using Kruskal-Wallis with Dunn’s post-test. Results were presented as means ± standard deviation (SD) or medians and interquartile range. *P*-values <0.05 were regarded as statistically significant.

## Results

### Oral Administration of Hsp65-Producing *L. lactis* Reduces *L. braziliensis* Lesion Size and Parasite Load

**Figure 1:**
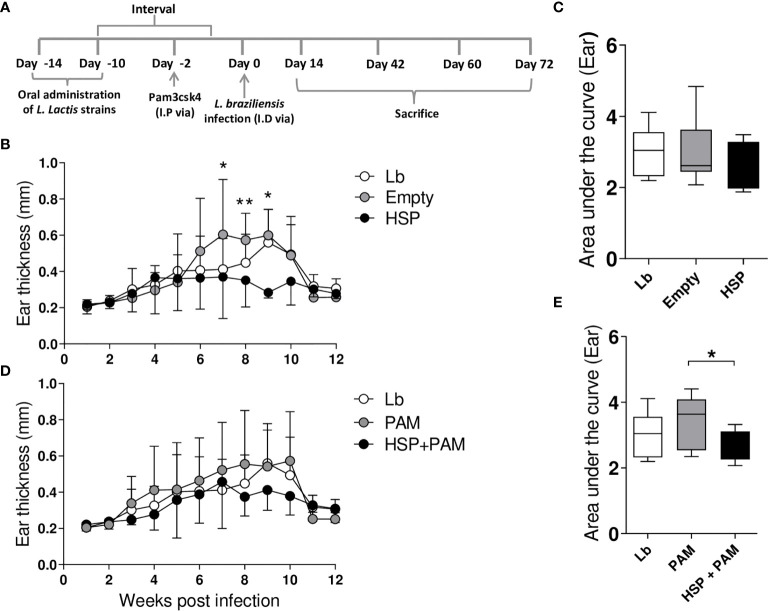
Experimental design and lesion development. Mice were treated with water (Lb and PAM), GM17 Medium containing empty vector-bearing *L. lactis* (Empty) or XM17 medium containing Hsp65-producing *L. lactis* (HSP and HSP+PAM) for 4 consecutive days, then infected with *L. braziliensis* metacyclic promastigotes *via* inoculation in the ear 10 days later. Two days prior to infection, the HSP+PAM and PAM groups received an intraperitoneal administration of PAM3CSK4. Lesion course was measured weekly using digital caliper. **(A)** Schematic drawing of the experimental protocol in BALB/c mice. **(B, C)** Ear thickness and area under the curve in Lb, Empty and HSP groups. The area under the curve was constructed using ear measurement data from each group. **(D, E)** Ear thickness and area under the curve of Lb, HSP+PAM and PAM groups. **(B, D)** Data are representative of means ± SD. **(C, E)** Data are representative of medians and interquartile range. Data are representative of four independent experiments using 10 animals per group. For nonparametric data, Kruskal-Wallis with Dunn’s post-test was used. (*) indicates P < 0.05 and (**) indicates P < 0.01 between Empty and HSP groups at 7, 8 and 9 weeks in **(B)**.

To evaluate the effects of Hsp65-producing *L. lactis*, lesions caused by *L. braziliensis* infection were monitored weekly (**Figure 1A**). The administration of Hsp65-producing *L. lactis* significantly decreased lesion size at weeks 7, 8 and 9 post-infection compared to mice that received the empty vector (**Figure 1B**), yet without significance in AUC values (**Figure 1C**). Interestingly, a balanced response was triggered in animals receiving PAM by i.p. route prior to infection in association with Hsp65 treatment, although no significant differences were found in lesion size compared to mice only receiving PAM (**Figure 1D**). However, our analysis of the area under the curve, which represented the evolution of disease, revealed significant reductions in lesion size in HSP+PAM mice compared to their respective controls (PAM) (**Figure 1E**).

**Figure 2:**
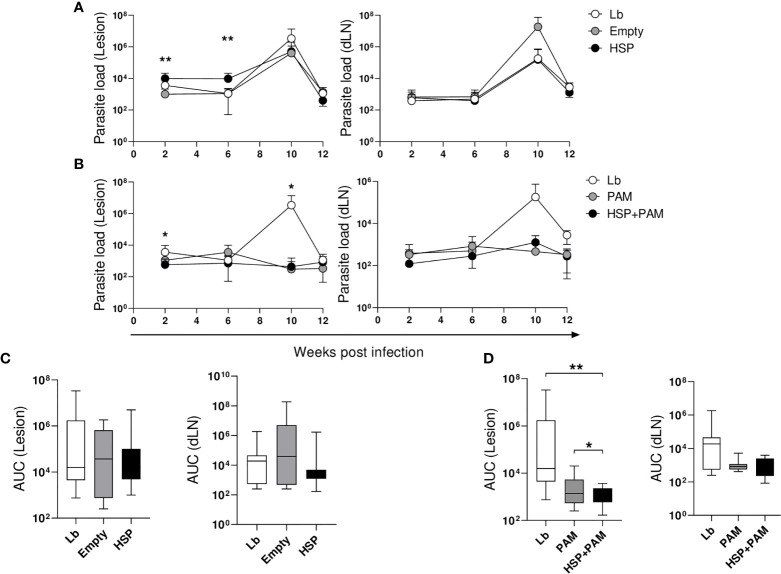
Kinetics of parasite load. Mice were treated with water (Lb and PAM), GM17 Medium with empty vector-bearing *L. lactis* (Empty) or XM17 medium containing Hsp65-producing *L. lactis* (HSP and HSP+PAM) for 4 consecutive days, then inoculated with *L. braziliensis* metacyclic promastigotes in the ear 10 days later. Two days prior to infection, the HSP+PAM and PAM groups received an intraperitoneal administration of PAM3CSK4. After euthanizing animals at 2, 6, 10 and 12 weeks post-infection, ears and draining lymph nodes were collected, cultured and parasite loads were then individually determined by limiting dilution assay. **(A)** Lb, Empty and HSP groups, **(B)** Lb, HSP+PAM and PAM groups, **(C)** area under the curve of Lb, Empty and HSP groups, **(D)** area under the curve of Lb, HSP+PAM and PAM groups. The area under the curve was constructed using parasite load data from each group. **(A, B)** Parasite numbers are represented as the means ± SD. **(C, D)** Parasite numbers are represented as medians and interquartile range. Data are representative of four independent experiments involving 10 animals per group. For nonparametric data, Kruskal-Wallis with Dunn’s post-test was used; (*) indicates P < 0.05 (between Lb and HSP groups at 6 weeks in **(A)**, Lb and HSP+PAM groups at 2 and 10 weeks in **(B)** and (**) P < 0.001 [between Empty and HSP groups at 2 and 6 weeks in **(A)**].

To assess whether lesion thickness was related to parasite numbers at the inoculation site and in the ear draining lymph nodes, parasite load was determined at 2, 6, 10 and 12 weeks post-infection. In the HSP group, parasite numbers in the ear were significantly higher at 2 and 6 weeks post-infection compared to animals receiving the empty vector (Empty) (**Figure 2A**). However, the HSP group was observed to control infection as evidenced by decreased parasite burden at 10 and 12 weeks post-infection, similarly to Lb and Empty groups (**Figure 2A**). No significant differences were seen in parasite burden in the draining lymph nodes (**Figure 2A**-right panel). Despite a lack of significance, the HSP+PAM group presented lower parasite numbers in both the lesion site and draining lymph nodes at all time points evaluated (**Figure 2B**). Lower parasite numbers were seen in the PAM group at all timepoints compared to the Lb group, providing evidence that lesion size is directly related to inflammatory response (**Figure 2B**). AUC analysis did not reveal any significant differences in the groups treated with HSP versus controls (Empty vector and Lb) (**Figure 2C**-left panel). However, AUC values were significantly lower in infected mice treated with HSP+PAM (**Figure 2D**-right panel). No significant differences in parasite load were observed among the groups in the draining lymph nodes (**Figure 2D**). No dissemination of parasites to other visceral organs, such as the spleen, liver or bone marrow, was observed in any of the experimental groups (data not shown).

### Oral Administration of Hsp65-Producing *L. lactis* Is Associated With Decrease of Inflammatory Cytokines

To evaluate changes in immune profile following the administration of Hsp65-producing *L. lactis*, a panel of cytokines was quantified at 2, 6, 10 and 12 weeks after *L. braziliensis* infection. Cells from the ear draining lymph nodes were obtained and re-stimulated *in vitro* with *L*. *braziliensis*, followed by cytokine quantification in culture supernatants by ELISA.

**Figure 3:**
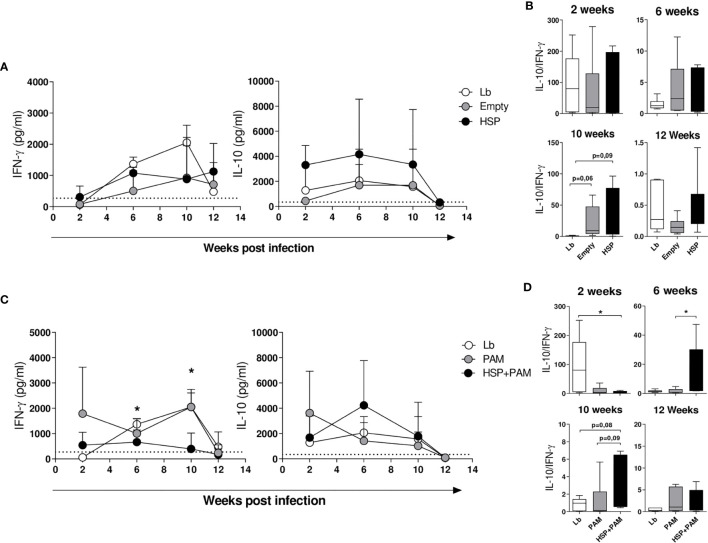
Cytokine production in draining lymph node cells following re-stimulation with *L. braziliensis.* Mice were treated with water (Lb and PAM), GM17 Medium with empty vector-bearing *L. lactis* (Empty) or XM17 medium containing Hsp 65-producing *L. lactis* (HSP and HSP+PAM) for 4 consecutive days, then inoculated with *L. braziliensis* metacyclic promastigotes in the ear 10 days later. Two days prior to infection, the HSP+PAM and PAM groups received an intraperitoneal administration of PAM3CSK4. After euthanizing animals at 2, 6, 10 and 12 weeks post-infection, draining lymph node cells were collected, cultured and restimulated *in vitro* with live promastigotes of *L. braziliensis* (1 cell:5 parasites). After 72h, the cell culture supernatants were collected to measure cytokines by ELISA. **(A)** IL-10 and IFN-γ production in Lb, Empty and HSP mouse groups. **(B)** IL-10:IFN-γ ratio in HSP, Empty and Lb mouse groups. **(C)** IL-10 and IFN-γ production in Lb, PAM and HSP+PAM mouse groups. **(D)** IL-10:IFN-γ ratio in Lb, PAM and HSP+PAM mouse groups. Dashed lines represent cytokine production in the absence of stimulus. **(A, C)** cytokine production is represented the mean ± SD. **(B, D)** IL-10:IFN-γ ratio is represented as medians and interquartile range. Data are representative of four independent experiments involving 10 animals per group. For nonparametric data, Kruskal-Wallis with Dunn’s post-test was used; (*) indicates P < 0.05 Lb and HSP+PAM at 6 and 10 weeks in **(C)**, PAM and HSP+PAM at 10 weeks in **(C)**.

At two weeks after infection, the Lb-infected groups treated or not with the empty vector or Hsp65 showed similarly low amounts of IFN-γ. At 6 weeks of infection, the levels of this cytokine increased across all groups, yet mice treated with the empty vector or Hsp65 presented relatively constant levels of IFN-γ compared to Lb; however, the latter group exhibited a consistent increase in IFN-γ levels until 10 weeks post-infection, followed by a decline at 12 weeks (**Figure 3A**). Regarding IL10 production, animals treated with Hsp65 presented higher levels of this cytokine at all timepoints, despite a lack of statistical significance. The ratio between these cytokines is depicted in **Figure 3B**, revealing higher IL-10 compared to IFN-γ in the groups treated with the empty vector and Hsp65 at 6 and 10 weeks after infection. Significant differences in the balance between IL-10 and IFN-γ production were observed at 10 weeks after infection.

Six weeks following infection, the HSP+PAM group presented significantly lower IFN-γ production than the PAM group (**Figure 3C**), which was also the case at 10 weeks after infection. Higher IL-10 concentrations were seen in the treated group (HSP+PAM) compared to PAM after 6 weeks of infection (**Figure 3C**). At 12 weeks post-infection, decreased levels of IFN-γ and IL-10 were observed in all groups, in contrast to increased levels of TGF-β observed across most groups, yet without significance (data not shown). IL-4 levels remained undetectable throughout infection (data not shown). With regard to the IL-10:IFN-γ ratio, predominantly higher IL-10 compared to IFN-γ was seen in the HSP+PAM group (**Figure 3D**), indicating that these animals achieved control of inflammation through increased IL-10 production.

### Administration of Hsp65-Producing *L. lactis* Induces a Higher Frequency of Regulatory T Cells in Mice

**Figure 4:**
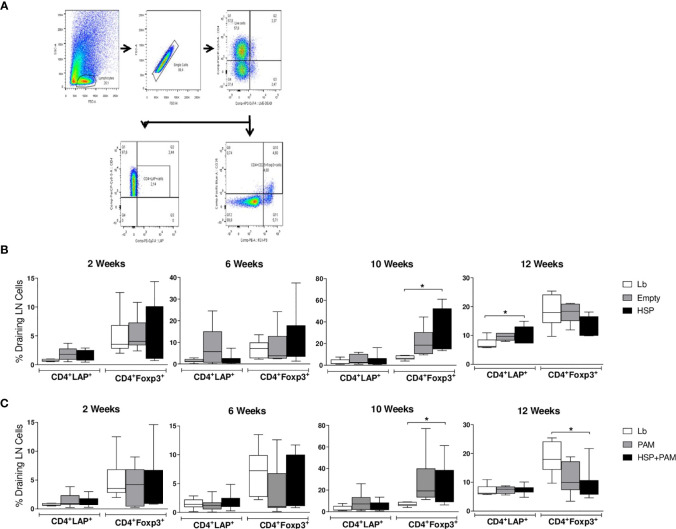
CD4^+^ LAP^+^ and CD4^+^ FoxP3^+^ regulatory T cell expression following re-stimulation with *L. braziliensis.* Mice were treated with water (Lb and PAM), GM17 medium with empty vector-bearing *L. lactis* (Empty) or XM17 medium containing *-*producing Hsp65-producing *L. lactis* (HSP and HSP+PAM) for 4 consecutive days, then inoculated with *L. braziliensis* metacyclic promastigotes in the ear 10 days later. Two days prior to infection, the HSP+PAM and PAM groups received an intraperitoneal administration of PAM3CSK4. After euthanizing animals at 2, 6, 10 and 12 weeks post-infection, draining lymph nodes were collected, cell suspensions were cultured with live *L. braziliensis* for 18h and stained using surface antibodies (anti-CD4 and anti-LAP) and intracellular molecules (anti-Foxp3). **(A)** Representative scatter plots of gating strategy. **(B)** Relative frequencies of CD4^+^ LAP^+^ and CD4^+^ Foxp3^+^ regulatory T cells in the draining lymph nodes of Lb, Empty and HSP groups. **(C)** Relative frequencies of CD4^+^ LAP^+^ and CD4^+^ Foxp3^+^ regulatory T cells in the draining lymph nodes of Lb, PAM and HSP+PAM groups. All frequencies of regulatory T cells are represented as medians and interquartile range. Data are representative of four independent experiments involving 10 animals per group. For nonparametric data, Kruskal-Wallis with Dunn’s post-test was used; (*) indicates P < 0.05.

The frequencies of CD4^+^ LAP^+^ T cells (expressing membrane TGF-β) and CD4^+^ Foxp3^+^ T cells were investigated by flow cytometry in draining lymph nodes. **Figure 4A** shows the strategy for gating the different subpopulations analyzed. At two weeks after infection, no significant differences were found (**Figures 4B, C**). At 10 weeks of infection, higher proportions of CD4^+^ Foxp3^+^ T cells were found in the HSP group compared to Lb controls (**Figure 4B**). A higher frequency of this subset was also found in the HSP+PAM group compared to Lb at this time point (**Figure 4C**). At 12 weeks of infection, the HSP group presented a significantly higher frequency of CD4^+^ LAP^+^ T cells compared to Lb, whereas significantly lower frequencies of CD4^+^ Foxp3^+^ T cells were seen in HSP+PAM mice at this timepoint (**Figure 4C**).

### Oral Administration of Hsp65-Producing *L. lactis* Reduces Inflammation at the Infection Site

**Figure 5:**
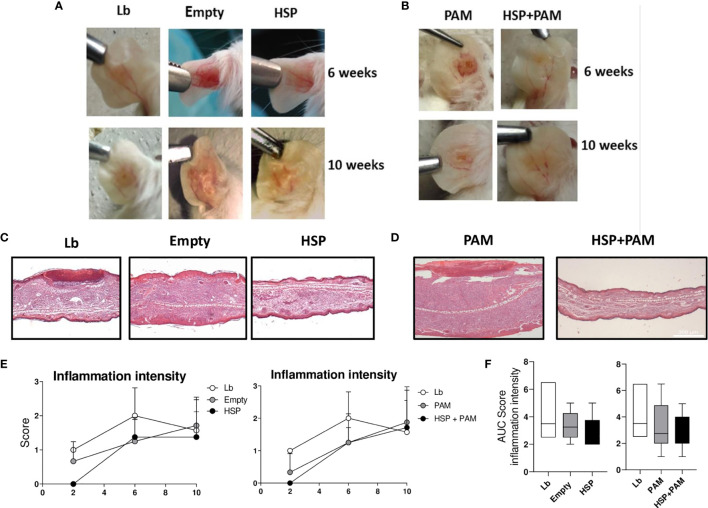
Lesion appearance, histological evaluation and quantitative score of inflammation intensity. Mice were treated with water (Lb and PAM), GM17 medium with empty-vector-bearing *L. lactis* (Empty) or XM17 medium containing Hsp65*-*producing *L. lactis* (HSP and HSP+PAM) for 4 consecutive days, then inoculated 10 days later with *L. braziliensis* metacyclic promastigotes in the ear. Two days prior to infection, the HSP+PAM and PAM groups received an intraperitoneal administration of PAM3CSK4. For nonparametric data, Kruskal-Walliis with Dunn’s post-test was used. **(A, B)** Representative lesion aspects at 6 and 10 weeks after infection in respective animal groups. **(C, D)** Ears were collected, fixed in 10% formaldehyde, processed and stained with hematoxylin and eosin. All sections were evaluated for histopathological changes, including inflammation intensity and the presence of inflammatory cell infiltrate. Scores for this parameter were determined according to intensity: Scores for each parameter were determined according to intensity: 0 (absence), 1 (presence of 1-25%), 2 (presence of 25-50%) and 3 (> 50%). Healthy animal ears were used as a negative control. **(E)** Kinetics of histological parameters in Lb, Empty and HSP groups (left panel) and Lb, PAM and HSP+PAM (right panel). Data are representative of means from each group. **(F)** Area under the curve analysis of histological scores obtained for each parameter evaluated. Quantitative scores are represented as medians and interquartile range. Data are representative of four independent experiments involving 8 animals per group. For nonparametric data, Kruskal-Wallis with Dunn’s post-test was used, no significance was found.

Macroscopic lesion observations and histological analysis were employed to evaluate inflammatory response. Decreased severity of *L. braziliensis* infection was observed by macroscopy in the HSP and HSP+PAM groups at 6 and 10 weeks of infection (**Figures 5A, B**).

Although no significant differences in lesion size were found after 6 weeks of infection (**Figures 1B, D**), more intense inflammation was detected in the Lb group compared to both Hsp65-treated groups, with reduced ulceration (**Figures 5C, D**). Histopathological analysis revealed higher inflammation intensity in the Lb control group compared to the Hsp65-treated groups (HSP, HSP+PAM) (**Figures 5E, F**), yet without statistical significance. Differences were also evident in the analysis of the area under curve shown in the right panel of **Figures 5E, F**.

### IL-10, TLR2, LAP Seem Important to the Regulation of Immunomodulatory Effects Mediated by the Oral Administration of Hsp65-Producing *L. lactis*

To investigate mechanisms underlying the immunomodulatory effects observed in Hsp65-treated BALB/c mice infected by *L. braziliensis*, we performed *in vitro* experiments that mixed cells from the mesenteric and draining lymph nodes in an attempt to mimic an *in vivo* setting. These experiments were performed *in vitro* due to convenience, as it is important to emphasize that BALB/c background knockout mice are not readily available. In addition, reports in the literature have shown that IL-10 and TLR2 knockout mice are highly resistant to infection by *Leishmania* spp. (37–39). We hypothesized that regulatory cells from Hsp65 treated mice would migrate to the lesion site, mitigating the inflammatory response mainly with respect to IFN-γ production.

**Figure 6:**
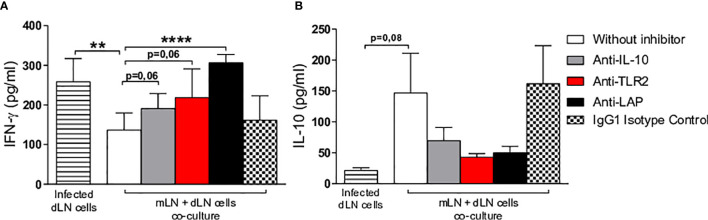
Evaluation of *in vitro* immune response. Macrophage-derived bone marrow cells from BALB/c mice were infected with *L. braziliensis* promastigotes at a ratio of 1:10 (cells:parasites) for 24 hours. Co-culturing was performed with infected macrophages, cells from the draining lymph nodes of animals at 10 weeks after infection and cells from the mesenteric lymph nodes of uninfected animals receiving Hsp65-producing *L. lactis* (collected after 10 days of treatment). Cells were incubated or not in the presence of anti-IL-10, anti-LAP or anti-TLR2. White and colored bars represent co-cultures of infected macrophages with draining and mesenteric lymph nodes cells, while striped bars represent infected draining lymph node cell cultures and hatched bars represent the IgG1 isotype negative control. Cytokine quantification of **(A)** IFN-γ and **(B)** IL-10 performed in cell supernatants. For parametric data, Student’s t-test was used: (**) P < 0.001; (****) P < 0.0001.

Cells from the mesenteric and draining lymph nodes were co-cultured in the presence or absence of neutralizing monoclonal antibodies (IL-10, LAP and TLR-2). Higher IFN-γ production was seen in the draining lymph nodes of infected mice compared to treated co-cultures of mesenteric and draining lymph nodes in the presence of anti-IL-10, anti-TLR2 or anti-LAP (**Figure 6A**). On the other hand, decreased levels of IL-10 were detected in co-cultures stimulated with infected macrophages with neutralizing monoclonal antibodies added in comparison to non-treated cultures, suggesting that these three components play an important role in regulating the immunomodulatory effects observed in our model (**Figure 6B**). Another important control group consisted of cells from mesenteric lymph nodes of Hsp65-treated mice co-cultured with cells from draining lymph nodes of infected mice with isotype IgG1 added; no differences were observed between this group and controls that did not receive an addition of monoclonal antibodies. In addition, despite a lack of statistical significance, higher levels of IL-10 were seen in co-cultures containing mesenteric lymph node cells from uninfected mice treated with Hsp65-producing *L. lactis* in the absence of monoclonal neutralizing antibodies compared to infected co-cultures.

### Oral Treatment With Hsp65-Producing *L. lactis* Modulates Inflammation at the Site of *Leishmania braziliensis* Infection

**Figure 7:**
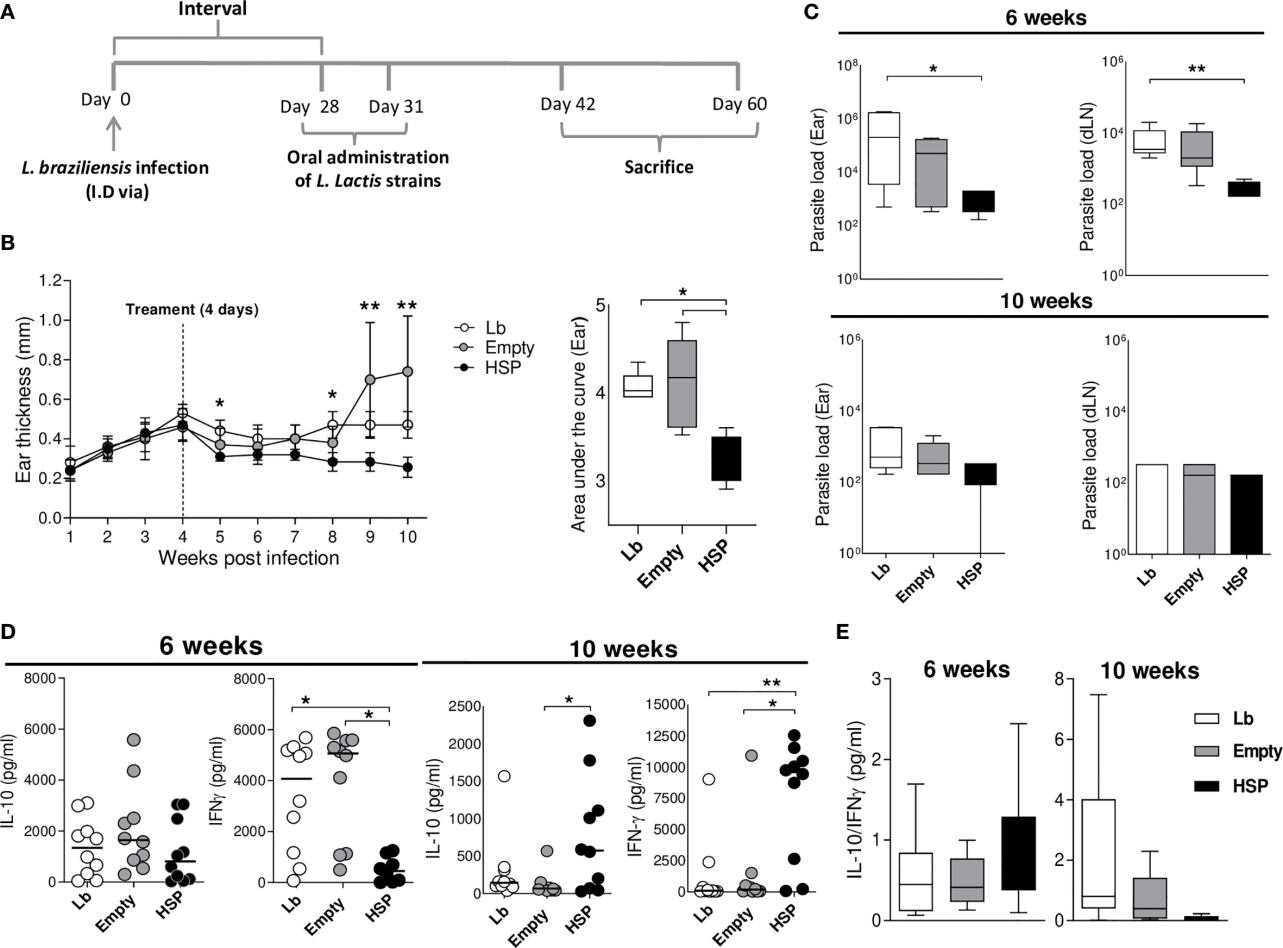
Oral treatment with *L. lactis*-Hsp65 post-infection. Mice were infected with *Leishmania braziliensis* metacyclic promastigotes in the ear, and four weeks after infection received oral treatment consisting of water (Lb), GM17 medium with empty-vector-bearing *L. lactis* (Empty) or XM17 medium containing Hsp65*-*producing *L. lactis* (Lb and HSP) for 4 consecutive days. **(A)** Experimental design of the post-infection treatment protocol in BALB/c mice. **(B)** Ear thickness was measured weekly. The area under the curve (right panel) was constructed using measurement data from each group. Data are representative of three independent experiments involving 10 animals per group. **(C)** Parasite load in the ear and draining lymph node lesions was determined by limiting dilution at 6 and 10 weeks post-infection. **(D)** Mice were euthanized at 6 and 10 weeks post-infection; draining lymph node cells were cultured and restimulated *in vitro* with live metacyclic promastigotes of *L. braziliensis* for 72 hrs. Cell culture supernatants were then collected for cytokine quantification by ELISA. **(E)** Ratio of IL-10/IFN-γ production. Data are representative of medians and interquartile range from each group. For nonparametric data, Krukal-Wallis with Dunns post-test was used. (*) *P* < 0.05 [between Lb and HSP groups at 5 and 8 weeks, **(B)**]; (**) *P* < 0.001 [between Empty and HSP groups at 9 and 10 weeks, **(B)**].

Since the experiments described above suggest that the oral administration of Hsp 65-producing *L. lactis* before infection decreases the inflammation caused by *L. braziliensis*, it was crucial to investigate these effects in a therapeutic strategy by administering recombinant *L. lactis* after infection. Accordingly, the oral administration of Hsp65-producing *L. lactis* was performed at 4 weeks after infection when lesions began to appear (**Figure 7A**).

To verify this immunomodulatory effects, ear thickness were measured weekly. Hsp65-treated animals showed smaller lesions during the entire period after treatment (at 5, 8, 9 and 10 weeks post infection) compared to those receiving an empty plasmid. This reduction was also reflected in the area under the curve of lesion size compared to controls (**Figure 7B**). In order to investigate whether Hsp65-producing *L. lactis* treatment had an effect on parasite load, ears and draining lymph nodes were collected to quantify parasite burden. Oral treatment with Hsp65-producing *L. lactis* led to a reduction in parasite burden at the infection site and in draining lymph nodes at 6 weeks after infection compared to *L. braziliensis*-infected controls. At 10 weeks of infection, no significant differences were seen between the experimental groups (**Figure 7C**). To evaluate changes in immune response profile after the oral administration of Hsp65-producing *L. lactis*, the quantification of inflammatory and anti-inflammatory cytokines was performed. Hsp65-treated animals showed a significant decrease in IFN-γ production compared to controls at 6 weeks of infection. However, at 10 weeks post-infection, significantly higher IL-10 and IFN-γ production was observed in animals treated with Hsp65-producing *L. lactis*-compared to the empty group (**Figure 7D**). No significant differences were observed between the experimental groups with regard to the IL-10:IFN-γ ratio (**Figure 7E**). No dissemination of parasites was observed in visceral organs in Lb, Empty and HSP.

## Discussion

Cutaneous leishmaniasis caused by *L. braziliensis* is characterized by chronicity, latency and the tendency to metastasize in the human host (41). The pathology observed in most CL cases results from an exacerbated inflammatory response. The modulation of this response is important for the control of disease and lesion healing.

Oral tolerance induced by dietary proteins has been shown to be an effective way to induce the development of antigen-specific regulatory cells (42, 43). The continuous oral administration of antigens was shown to generate regulatory T cells capable of suppressing the inflammatory response in experimental models of autoimmune disease (14, 15). Therefore, the induction of regulatory T cells, such as CD4^+^Foxp3^+^ and CD4^+^LAP^+^, as well as the production of anti-inflammatory cytokines, e.g., IL-10 and TGF-β, appears to be crucial to the induction of oral tolerance (43).

Most studies attempting to induce oral tolerance by producing an anti-inflammatory response to decrease inflammation in autoimmune disease have intravenously administered the same antigen that originally induced the pathology [for instance, oligodendrocyte myelin glycoprotein in experimental encephalomyelitis and type I diabetes] (44, 45). However, it has been confirmed that antigens unrelated to the original pathology can also inhibit inflammatory processes *via* a phenomenon known as “bystander” suppression. This experimental approach is useful when the target antigen triggering the pathology is unknown (46). This suppression is associated with oral tolerance due to inhibitory effects against other antigens, possibly resulting from the action of regulatory T cells producing non-specific cytokines (47–49). In the search to identify these antigens, emphasis has been placed on heat shock proteins as these proteins participate in many inflammatory events (50, 51), are highly conserved among species and exert regulatory activity over the immune system. Hsps are considered immunodominant autoantigens as these molecules are easily recognized by the repertoire of autoimmune lymphocytes under physiological conditions (52). The anti-inflammatory role of Hsp in inflammatory and autoimmune diseases has been demonstrated in several models, e.g., diabetes, arthritis, atherosclerosis and multiple sclerosis (32, 53–55). The oral administration of recombinant Hsp65 has been shown to induce tolerance and protect rats against adjuvant arthritis (56), as well as mice against atherosclerosis (32, 57). The present study represents the first attempt to use Hsp65 to mitigate an inflammatory disease caused by a protozoan species, such as *Leishmania.*

In this study, we showed that the oral administration of Hsp65-producing *L lactis* prior to *L. braziliensis* infection attenuated lesion development and decreased inflammatory aspects, preserving mouse ear tissue in the experimental groups (HSP and HSP+PAM), despite the absence of significant differences in histological scores. Surprisingly, in a set of experiments involving the Lb, Empty and HSP groups, significant differences in lesion size and parasite load were observed between the Empty and HSP groups, suggesting that the administration of bacteria alone before infection aggravated the inflammation associated with disease. Accordingly, we may hypothesize that this bacterium also could induce regulatory T cells earlier than HSP, which could explain the observed outcome. In the set of experiments involving PAM or treatment with Hsp65 after infection, differences were noted between the Lb and HSP-treated groups: PAM induced a strong inflammatory response capable of parasite elimination, while HSP downmodulated this exacerbated inflammation. Another interesting point is that Hsp65 treatment after infection also led to decrease disease severity, clearly suggesting that Hsp65 represents a potential alternative therapeutic strategy. In addition, no parasite dissemination was observed, regardless of when Hsp65-producing *L. lactis* was orally administered (prior to or after infection). It is also important to emphasize that when HSP was administered after infection, PAM was not employed as the inflammation provoked by the parasite was sufficient to trigger an anti-inflammatory response elicited by recombinant *L. lactis.*

In experimental control groups (Lb, Empty and PAM), extensive tissue destruction resulting from intense cellular immune response activity was observed, along with high IFN-γ production characteristic of the cutaneous leishmaniasis model (58). However, in draining lymph node cells from mice receiving Hsp65-producing *L. lactis*, decreased IFN-γ production and increased IL-10 secretion was observed, albeit despite a lack of significance. At the beginning of infection, it is possible that decreased levels of IFN-γ are responsible for a transitory enhancement in the number of parasites; nonetheless, this was evidently controlled and inflammation was modulated at later times of infection.

Rezende et al. (16), using the same protocol as that employed in the present study, demonstrated that the induction of oral tolerance prevented the development of experimental encephalomyelitis *via* increased IL-10 production by regulatory T cells present in the mesenteric lymph nodes and spleen.

IL-10, an anti-inflammatory cytokine produced by a variety of cells, has been shown to inhibit the Th1-type response induced by *Leishmania* sp. infection (59). Despite being associated with parasite persistence, IL-10 acts as an important immunoregulator in tissue remodeling during the healing process, minimizing the damage caused by an exacerbated immune response and associated inflammatory cytokine production, notably IFN-γ (60–64). Although significant differences between IL-10 and IFN-γ production were not observed among the experimental groups herein, our results showed a predominance of IL-10 production compared to IFN-γ in mice treated with Hsp (HSP or HSP+PAM), which contributed to decreased lesion severity, as well as the absence of ulceration. In addition, the production of IFN-γ also appeared to have contributed to the control of parasite load in these animals. The treatment of Hsp65 after infection resulted in low levels of IFN-γ at 6 weeks after infection comparing to Lb and Empty control groups. It is possible that decreased amounts of IFN-γ were sufficient to eliminate parasites and that IL-10 concentrations, despite no significant differences between other groups, controlled inflammation, which led to better *L. braziliensis* infection outcomes. Although higher levels of IL-10 were seen at 10 weeks after infection in the Lb group, at this point exacerbated inflammation and high numbers of parasites worsened lesions caused by *L. braziliensis* infection.

As our results concerning regulatory T cells were variable throughout the experiments, a clear pattern did not emerge. We intended to investigate these cell populations in ear lesions in situ; unfortunately, the concentrations of cells in mouse ears were very limited, and small numbers of regulatory T cells were identified. The lack of significant differences between empty and Hsp-65 producing *L. lactis* lymph node cultures seems to suggest that *L. lactis* alone may also influence the induction of regulatory T cells in the gut, yet not at the intensity seen in the HSP group.

The fact that Hsp increases IL-10 induction has already been evidenced in some experimental studies. In an atherosclerotic model, the oral administration of Hsp65 was shown to increase IL-10 levels at the site of inflammation, along with reduced inflammatory activity (32). Another study showed that disease pathogenesis, as well as endothelial damage, were attenuated by the effects of increased IL-10 and a reduction in IFN-γ induced by oral tolerance to Hsp65 (33). Consistent with the important role of IL-10, Wieten et al. demonstrated that immunization with Hsp70 in mice 10 days before the induction of arthritis inhibited the clinical and histological signs of disease *via* the production of IL-10 (65). In a model of colitis, the administration of *L. lactis* Hsp65 prevented a reduction in IL-10 levels in colon tissue, which was shown to be critical to immunoregulation (17). A similar effect was observed in experimental models of encephalomyelitis and arthritis, in which the oral administration of Hsp65-producing *L. lactis* was shown to prevent disease development in association with increased IL-10 production (16, 66), as well as in an IL-10-dependent fashion (66).

An experimental model of cutaneous leishmaniasis demonstrated that the presence of Foxp3^+^ Treg cells in the epidermis was associated with a controlled immune response that limited tissue pathology (67, 68). Although no significant differences were found in the frequency of CD4+Foxp3+ T cells between all groups evaluated at the beginning of infection (2 and 6 weeks post-infection), we found that the oral administration of Hsp65-producing *L. lactis* increased the frequency of CD4^+^Foxp3^+^ (at 10 weeks of infection) in cultures of draining lymph node cells, indicating that regulatory T cells may be involved in mitigating the inflammation caused by *L. braziliensis*. Several experimental models of chronic inflammatory disease, such as atherosclerosis (32, 69), rheumatoid arthritis (65) and diabetes (70), have shown that the administration of Hsp65 induced Treg cells.

Recently, two studies using Hsp65-producing *L. lactis* demonstrated that pre-treatment with Hsp65 prevented the development of colitis (17) and arthritis (66) in mice by inducing CD4^+^Foxp3^+^ and CD4^+^LAP^+^ Treg cells in a TLR2-dependent manner; in addition, reduced inflammatory cytokine production in the colon (17) and draining lymph nodes (66) were seen. The oral administration of Hsp60, which binds to TLR2, was shown to inhibit the production of TNF-α and IFN-γ, and increase IL-10 secretion by T cells, which acts on the maintenance and function of Treg cells *via* TLR2 (28).

The intestinal mucosa is known to be a privileged site for the generation of surface-expressing LAP^+^ Treg cells (23–25). Here we observed that animals receiving Hsp presented an increased frequency of CD4^+^LAP^+^ T cells (after 12 weeks of infection), which suggests the role of this T cell type in our model. Results from an experimental model of autoimmune encephalomyelitis indicate that the high numbers of CD4^+^LAP^+^ Treg cells found in mesenteric lymph nodes can be induced in the intestinal mucosa following the administration of Hsp65-producing *L. lactis*, which then migrate to secondary lymphoid organs (16). A similar effect could be occurring in the present model, since CD4+Foxp3+ and CD4+LAP+ regulatory T cells were also found in the draining lymph nodes at *L. braziliensis* infection lesion sites.

Our *in vitro* experiments that mixed mesenteric and draining lymph node cells demonstrated that the production of IFN-γ and IL-10 is dependent on TLR2, LAP and IL-10, since the monoclonal antibodies neutralizing these molecules induced increased IFN-γ and decreased IL-10 production, confirming previous data found in models of colitis and arthritis (17, 66).

Resistance to infection is a function of the immune system, which acts by detecting, neutralizing and destroying pathogens, thereby reducing the pathogen burden. Both innate and adaptive immune systems contribute to infection resistance (71). Resistance to infection was seen in the group of animals treated with PAM, as evidenced not only by the killing of parasites, but also the destruction of ear tissue resulting from the exacerbated immunopathology caused by the parasite and PAM. A favorable immune response is a result of the balance between an acceptable level of immunopathology and pathogen elimination (72). Indeed, the factor required to reduce the burden of the pathogen is the same that causes immunopathology (73). Tolerance to the pathogen reduces the negative impact of an infection on the host without directly affecting parasite load (73–75).

Our data in the HSP and HSP+PAM groups leads us to believe that the administration of Hsp65-producing *L. lactis* is capable of controlling tissue damage by way of mechanisms related to infection tolerance, a host defense strategy that reduces the negative impact of tissue destruction resulting from infection. Unlike resistance mechanisms, tolerance does not directly bear on parasite load; rather it mitigates the damage caused to the host by pathogens, or host immune response (76).

Another remarkable finding was the role played by TLR2 in the regulatory effect mediated by Hsp65. Hsp65 and PAM are both ligands of TLR2. However, they trigger distinct effects when administered. It is plausible that strong agonists, such as PAM, would promote an inflammatory response in innate cells (monocytes and DCs), whereas analogues of endogenous ligands, such as Hsp65, trigger balanced mix of cytokines.

Our results demonstrate the ability of Hsp65-producing *L. lactis* to mitigate the effects of inflammation caused by *L. braziliensis* infection through the evaluation of anti-inflammatory cytokine production and the expansion of regulatory T cells in the draining lymph nodes of BALB/c mice. We therefore suggest that Hsp65-producing *L. lactis* can be considered as a potential candidate for treatment not only in autoimmune disease, but also in chronic inflammatory diseases caused by pathogens.

## Data Availability Statement

The raw data supporting the conclusions of this article will be made available by the authors, without undue reservation.

## Ethics Statement

The animal study was reviewed and approved by CEUA (Committee for Ethical Animal Use in Experimentation No 006/2013 IGM/Fiocruz-Bahia.

## Author Contributions

PVG - carrying out the experiments and writing the manuscript. CMA, IVN, BCG, and RT- carrying out the experiments WLCS - histopathological analysis. VAA – constructed and provided us with recombinant Lactococcus lactis bacteria and designed the experiments. NMT- statistical analysis and layout of the figures. JSR – design of experiments and discussion of results. TUM - design the experiments and controls and writing the manuscript AMCF - design the experiments, analysing the data, and writing the manuscript. CIB - coordinate the study, design the experiments, supervise them, discuss the results, and writing the manuscript. All authors contributed to the article and approved the submitted version.

## Funding

This study was supported by CNPq grants numbers 428371/ 2018-3 and 304876/2019-4. PVG received a pos-doc fellowship from Fundação Oswaldo Cruz, Programa Inova. VAA, TUM, AMCF, and CIB are senior investigators of CNPq. This study is financed in part by the Coordenação de Aperfeiçoamento de Pessoal de Nıvel ´ SUperior (CAPES)-Brazil, Finance Code 001.

## Conflict of Interest

The authors declare that the research was conducted in the absence of any commercial or financial relationships that could be construed as a potential conflict of interest.
